# How to Use CA-125 More Effectively in the Diagnosis of Deep Endometriosis

**DOI:** 10.1155/2017/9857196

**Published:** 2017-06-04

**Authors:** Marco Aurelio Pinho Oliveira, Thiers Soares Raymundo, Leila Cristina Soares, Thiago Rodrigues Dantas Pereira, Alessandra Viviane Evangelista Demôro

**Affiliations:** Department of Gynecology, State University of Rio de Janeiro, Rio de Janeiro, RJ, Brazil

## Abstract

Deep infiltrative endometriosis (DIE) is a severe form of the disease. The median time interval from the onset of symptoms to diagnosis of endometriosis is around 8 years. In this prospective study patients were divided into two groups: cases (34 DIE patients) and control (20 tubal ligation patients). The main objective of this study was to evaluate the performance of CA-125 measurement in the menstrual and midcycle phases of the cycle, as well as the difference in its levels between the two phases, for the early diagnosis of DIE. Area Under the Curve (AUC) of CA-125 in menstrual phase and of the difference between menstrual and midcycle phases had the best performance (both with AUC = 0.96), followed by CA-125 in the midcycle (AUC = 0.89). The ratio between menstrual and midcycle phases had the worst performance. CA-125 may be useful for the diagnosis of deep endometriosis, especially when both are collected during menstruation and in midcycle. These may help to decrease the long interval until the definitive diagnosis of DIE. Multicentric studies with larger samples should be performed to better evaluate the cost-effectiveness of measuring CA-125 in two different phases of the menstrual cycle.

## 1. Introduction

Endometriosis is characterized by the development of endometrial tissue outside the uterine cavity. Deep infiltrative endometriosis (DIE) is a severe form of the disease and can affect many anatomical structures like the uterosacral ligaments, parametrium, bladder, and bowel. Dysmenorrhea is the most common symptom. Other symptoms include dyspareunia, low back pain, dyschezia, and dysuria [1].

Although the clinical manifestations of endometriosis are not specific, it is known that women with endometriosis experience significantly more gynecological, urological, and bowel symptoms than women without endometriosis. Adequate anamnesis and physical examination favor the diagnosis and, in some cases, they may also help suggesting the probable site of the disease [2].

Transvaginal and transrectal ultrasound, pelvic magnetic resonance imaging (MRI), colonoscopy, and cystoscopy may help in the diagnosis of DIE. Even with the advance in image exams, the median time interval from the onset of symptoms to diagnosis of endometriosis, including DIE, is around 8 years [3].

Many studies have been conducted to evaluate the feasibility of using serum CA-125 in the diagnosis of patients with clinical suspicion of endometriosis. They present different results, mainly in relation to their sensitivity [4–7]. The difficulty of its use is to establish an appropriate cutoff value, since the current value (35 IU/mL) is the reference for ovarian cancer of epithelial origin. CA-125 levels appear to fluctuate during different phases of the menstrual cycle, especially during menstruation [8]. The use of the difference of CA-125 levels between menstruation and the midcycle for the diagnosis of endometriosis is still poorly explored and this may be a useful information in the evaluation of patients with clinical and/or radiological suspicion of endometriosis. CA-125 levels tend to be higher during menstruation, possibly due to the increased inflammatory activity of the endometriotic cells [8].

The main objective of this study is to evaluate the performance of CA-125 measurement in the menstrual and midcycle phases of the cycle, as well as the difference in its levels between the two phases, for the early diagnosis of DIE.

## 2. Patients and Methods

A prospective study was conducted in women who were referred to outpatient gynecology clinic or at Endometriosis Treatment Center in Pedro Ernesto University Hospital (Rio de Janeiro State University) between January 2012 and January 2016. The main indications for surgery were deep endometriosis (case group) and tubal ligation (control group). All patients with clinical suspicion of endometriosis had an MRI before surgery. Surgical procedures were performed by laparoscopy. The study was approved by the Ethics and Research Committee of the Hospital. Informed consent was obtained from all participants.

A total of 54 patients were included, 34 with deep infiltrative endometriosis (DIE) and 20 for tubal ligation. Two serum samples of CA-125 were collected during the preoperative period: one during menses (between the 2nd and 4th days of the menstrual cycle) and the other in the midcycle (between the 13th and 15th days of the menstrual cycle). Both dosages were made no more than 3 months prior to surgery. Serum CA-125 concentrations were measured by an immunoradiometric kit using M11 specific monoclonal antibody (Centocor, Malvern, PA, USA).

Patients with a previous diagnosis of endometriosis who had used any type of hormonal medication (including contraceptive pills) in the last three months before CA-125 collection or with diagnosis of adenomyosis by ultrasound or MRI were excluded from the study. In patients with endometriosis, complete resection of the disease was performed during laparoscopy.

After laparoscopy, patients were divided into two groups: confirmed endometriosis and without endometriosis. The diagnosis was suspected by visual identification of the lesions and confirmed by histopathological analysis, considering the gold standard. *χ*^2^ test was used to analyze proportions and the nonparametric Mann-Whitney *U* test and Wilcoxon signed-rank test were used to compare between and within groups, respectively. Diagnostic performance of CA-125 was obtained by calculating sensitivity (Sn), specificity (Sp), positive likelihood ratio (LR+), negative likelihood ratio (LR−), and Area Under the Curve (AUC). Statistical analyses were performed using MedCalc for Windows, version 15.0 (MedCalc Software, Ostend, Belgium). A value of *p* < .05 was considered statistically significant.

## 3. Results

The study group included 34 patients with histological diagnosis of DIE and the control group consisted of 20 patients without visual diagnosis of endometriosis at the time of laparoscopy. Their baseline characteristics are outlined in Table 1. The groups were similar according to age but gravidity was significantly higher in the tubal ligation group, as expected.

**Figure 1:**
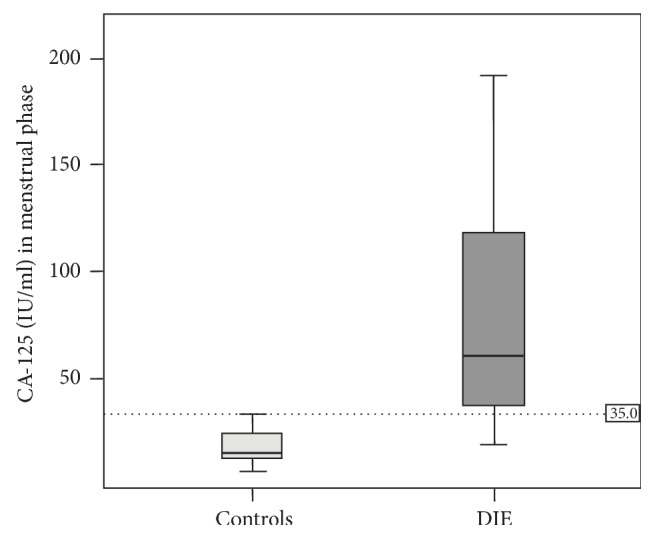
Boxplot showing the distribution of CA-125 between controls and patients with deep infiltrative endometriosis (DIE) in the menstrual phase. Two extreme values (324 and 426 IU/mL) were removed from the chart in the DIE group to obtain a better scaling of the graphic.

Serum CA-125 values were significantly higher in patients with DIE than in controls in both phases of the cycle. Median CA-125 in the menstrual phase were 65.8 IU/mL (range 20.5–426.0 IU/mL) in DIE group and 16.6 IU/mL (range 8.0–35.9 IU/mL) in controls. Median difference between DIE group and controls in the menstrual phase was 49.2 (*p* < .001). All but one patient in the control group had a CA-125 lower than the usual cutoff value of 35 IU/mL in the menstrual phase and most patients with DIE (85%) had values above this threshold (Figure 1).

**Figure 2:**
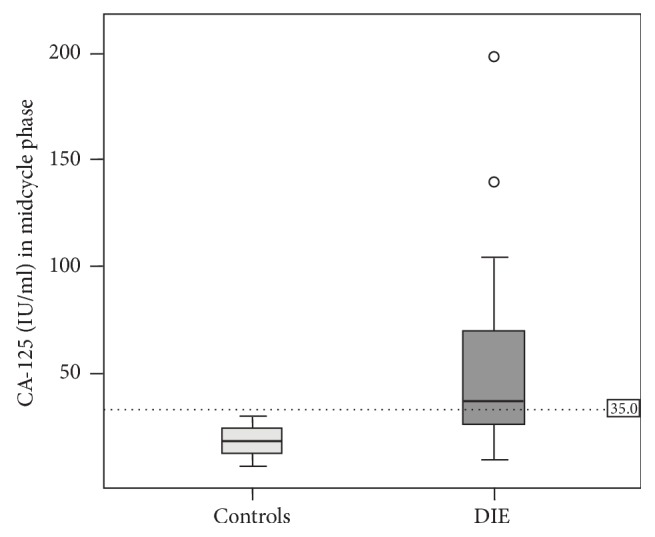
Boxplot showing the distribution of CA-125 between controls and patients with deep infiltrative endometriosis (DIE) in the midcycle phase. ○ = outliers.

Serum CA-125 values were lower in midcycle than in menstrual phase, in both groups. Median CA-125 in the midcycle phase were 39.5 IU/mL (range 11.9–200.0 IU/mL) in DIE group and 16.4 IU/mL (range 5.4–30.8 IU/mL) in controls. Median difference between DIE group and controls in the menstrual phase was 23.1 (*p* < .001). All patients in the control group had a CA-125 lower than 35 IU/mL in the midcycle phase and only 53% patients with DIE had values above this threshold (Figure 2).

We also evaluated the performance of the difference of CA-125 between menstrual and midcycle phases (ΔCA-125) and the ratio (in percentage of increase) of CA-125 between midcycle and menstrual phases. The median of ΔCA-125 in controls was 1.95 (CI 95% 1.21–3.71) and in DIE patients 18.20 (CI 95% 13.76–29.84). This difference was statistically significant (*p* < .0001). The median of the percentage increase of CA-125 in controls was 13.6% (CI 95% 8.2%–24.6%) and in DIE patients 58.7% (CI 95% 45,9%–71.9%). This difference was statistically significant (*p* < .0001).

Using CA-125 in menstrual phase, with a cutoff of 35 IU/mL, Sn, Sp, LR+, and LR− were, respectively (with CI 95%), 95% (75.1% to 99.8%), 85.3% (68.9% to 95.0%), 6.46 (2.86 to 14.61), and 0.06 (0.01 to 0.40). Using also the same cutoff level in the midcycle phase, Sn, Sp, LR+, and LR− were, respectively (with CI 95%), 52.9% (35.13% to 70.22%), 100% (83.16% to 100.00%), 2.12 (1.49 to 3.04), and 0.0.

**Figure 3:**
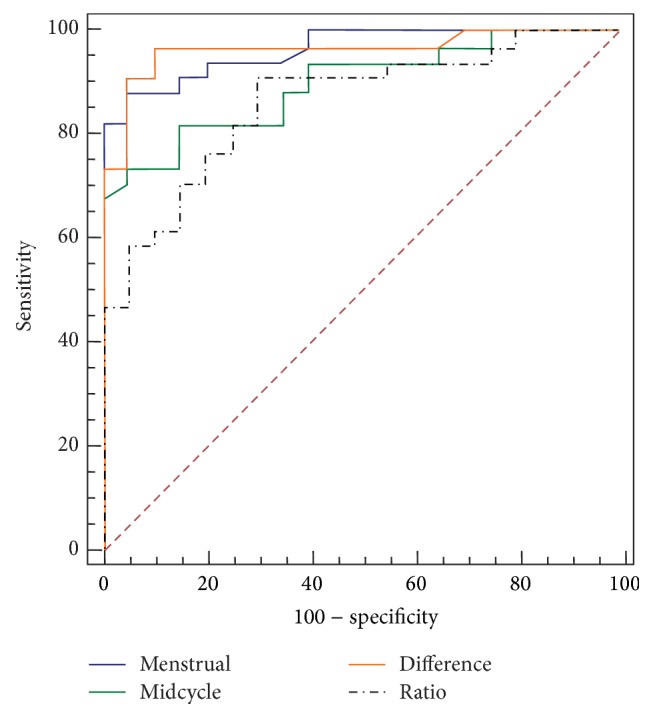
ROC curves for CA-125 in menstrual and midcycle phases as well as for the difference and ratio between the two phases.

We compared AUC for CA-125 in menstrual and midcycle phases as well as for the difference and ratio between the two phases (Figure 3).

AUC of CA-125 in menstrual phase and of ΔCA-125 between menstrual and midcycle phases had the best performance (both with AUC = 0.96), followed by CA-125 in the midcycle (AUC = 0.89). The ratio between menstrual and midcycle phases had the worst performance (Table 2).

Using CA-125 cutoff of 35 IU/mL, five women with DIE had negative tests in both phases of the cycle (14% false-negative for DIE). There were no cases of both tests positive in the control group. Only one woman in the control group had a positive test in menstruation, but it was negative in midcycle (Table 3).

The best cutoff point in AUC for ΔCA-125 was the 8.5 value for the difference between CA-125 in menstruation and in midcycle. When menstrual serum CA-125 levels were less than 35 IU/mL in women with DIE, four had ΔCA-125 above 8.5 IU/mL. The only patient in the control group with serum CA-125 level in menstruation > 35 IU/mL had a ΔCA-125 less than 8.5 IU/mL. The specificity of this test was 100%.

## 4. Discussion

Early diagnosis of DIE is of utmost importance. There is usually a large diagnostic delay, reaching 6 or more years, even in large centers [9]. This delay occurs even in patients with deep endometriosis who have more severe disease. Despite the advances in imaging methods, such as transvaginal ultrasound and magnetic resonance imaging (MRI), both with bowel preparation, the situation is still far from ideal because they are operator dependent [10, 11].

An efficient serum biochemical marker would be helpful, as it could potentially make the screening more accessible and could also be easily standardized. CA-125 is the serum biomarker that has been more extensively studied in the diagnosis of endometriosis, but it had not reached its full potential. Other serum markers such as CA19-9, interleukins 6, 8, and 10, and tumor necrosis factor alpha have also already been studied [12, 13]. In comparative studies, those markers were not superior to CA-125 in relation to diagnostic performance in endometriosis. However, no serum biomarkers have been validated for a noninvasive diagnostic test with adequate sensitivity and specificity [13, 14].

The purpose of our study was to improve the diagnostic performance of CA-125 by measuring the difference between the values of this biomarker collected in menstruation and in midcycle. The rationale is that, in women with endometriosis, the CA-125 would reach its highest level during menstruation due to the most marked inflammatory process. Fluctuations in CA-125 levels during menstruation seem to occur by endometrial desquamation during menstruation and consequent breakdown of tissue-hematological barrier for a short period [8]. With two measurements, it is possible to evaluate the performance of CA-125 during menstruation and midcycle and to calculate the difference between these two moments in the cycle.

Some authors have published data on serum CA-125 concentrations in spontaneous and stimulated menstrual cycles and have shown that its levels fluctuate during the menstrual cycle [15, 16].

In our study, we observed that CA-125 concentrations during menses were higher when compared to CA-125 levels in midcycle in patients with endometriosis. Similar finding was described by Kafali et al., but blood samples were collected three months after surgery, with patients already treated. It is known that resection of endometriosis is associated with reduction in CA-125 levels [8]. It is more useful for diagnosis of endometriosis to evaluate CA-125 before surgery than after surgical treatment.

We did not find in the literature a similar study evaluating the difference (additive scale) between menstrual and midcycle CA-125 values. Koninckx et al. evaluated the ratio between two consecutive cycles with blood samples taken during menstruation and 7 days later, during the midfollicular phase. The CA-125 median value of DIE group during menstrual phase was 84 IU/mL and during midfollicular phase was 55 IU/mL. Although the mean difference was high (29 IU/mL) they used the menstrual/midfollicular ratio to improve the diagnosis but they did not find any diagnostic potential of its use [17]. In our study, we found that the ratio (increase in percentage) had the worst performance. The difference between menstrual and midcycle phases had a superior performance than the ratio.

Another interesting finding was that the positivity of CA-125 (>35 IU/mL) in the midcycle phase increased the probability of the diagnosis of DIE to 100% in our sample. No patient in the control group (tubal ligation) had CA-125 levels higher than 35 IU/mL outside menstruation. Therefore, we could use CA-125 outside menstruation to rule in deep endometriosis, as we had a specificity of 100% in our sample. Caution should be taken because of the small sample size since the 95% confidence interval is quite large (83.16% to 100.00%).

We also found higher CA-125 levels during menstruation than during the nonmenstruation in patients with DIE when compared to patients in the control group. Chapron et al. found a similar performance in patients with deep endometriosis. Serum CA-125 levels were significantly higher in DIE group (55.2 ± 68.7 U/mL) compared to controls (22.5 ± 25.2 U/mL; *p* < .001). Some hypotheses tried to explain this increase: presence of blood and eutopic endometrial tissue into the peritoneal cavity due to retrograde menses, enlarged surface of endometrial tissue, and inflammatory reaction in the endometrial foci [18]. Bon et al. suggested that CA-125 released from the endometrium can have access to the lymphatics and the circulation [19].

**Figure 4:**
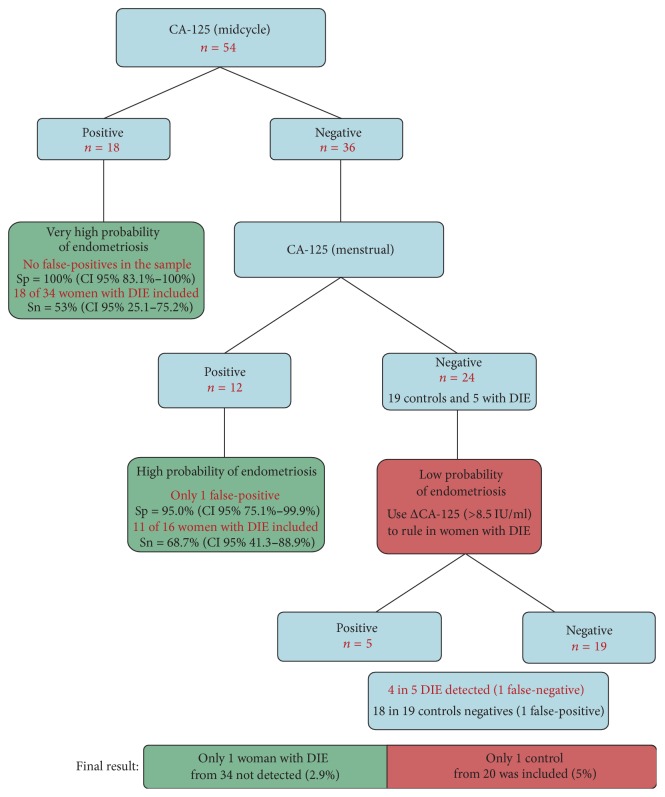
Suggested flow diagram to use CA-125 more effectively in the diagnosis of deep endometriosis.

The performance of CA-125 in the menstrual and midcycle phases was quite similar as evaluated by the AUC. What would be the advantage of using both measures instead of just one? As shown in the flowchart (Figure 4), the first potential advantage of using CA-125 in two different phases of the cycle is the possibility of identifying a high CA-125 level in the middle of the cycle. Of the 18 patients with positive midcycle CA-125, all were diagnosed with DIE. When midcycle CA-125 is negative, measuring menstrual CA-125 could be helpful. If it is positive, there is a high probability of endometriosis. If it is negative, the probability of endometriosis is low, but some women with DIE have negative CA-125 in both phases (5 in 34 in our sample, 14,7%). In this scenario, one additional advantage is being able to calculate the difference of the measurements of the CA-125. Even when the two measures were negative, using ΔCA-125, 4 out of 5 patients (80%) with DIE were positive (>8.5 IU/mL). In the control group, only 1 patient in 19 was positive (false-positive of 5,2%).

One strength of our study was related to the exclusion criteria. We excluded women who were using oral contraceptives, with previous diagnosis of endometriosis or with the diagnosis of adenomyosis by MRI or transvaginal ultrasound. Most studies do not exclude patients on contraceptive pills or with adenomyosis. The use of contraceptive pills can decrease CA-125 levels [20] and the presence of adenomyosis can increase it [21]. These conditions may mislead the interpretation of CA-125 in the diagnosis of endometriosis. However, those excluding criteria were the main reason for the relatively small sample size, compared to other previous published studies. It is important to mention that the results of our study must be validated in other series (cross validation), since we know that the performance of a test is overrated for any sample.

One of the limitations of our study is that not all the spectrum of disease was included. We did not include cases with only suspected peritoneal endometriosis or with subtle DIE lesions (*p* ex lesions less than 2 cm affecting only the uterosacral ligament). The performance of the double measurement of CA-125 may be lower when including those patients. However, even patients with maybe obvious DIE are frequently not diagnosed by nonspecialized centers. Hudelist et al. evaluated 171 women with histologically proven endometriosis. A delay of 10.3 years was observed for patients with extensive DIE, unfortunately similar to superficial/peritoneal endometriosis (10.5 years, *p* = .87) [22]. Therefore, ΔCA-125 still might be helpful to make an early diagnosis of many patients with DIE, potentially decreasing the devastating effects of this disease.

In this study, we can conclude that CA-125 may be useful for the diagnosis of deep endometriosis, especially when both are collected during menstruation and in midcycle. These findings may help to decrease the interval between the first complaints and the definitive diagnosis of deep endometriosis. Multicentric studies with larger samples should be performed to understand better the cost-effectiveness of measuring CA-125 in two different phases of the menstrual cycle for the early diagnosis of DIE.

## Figures and Tables

**Table 1:** Baseline characteristics of patients with dosages of CA-125 in menstruation and in the midcycle.

Variables	* Controls* (*n* = 20)	* DIE* (*n* = 34)	*p* value
Age (years)	33.7 (±8.0)	34.2 (±5.1)	.82^b^
Gravidity			
0	0	23 (69.7%)	<.001^*∗*c^
1	6 (30%)	6 (18.2%)	
2	11 (55%)	3 (9.1%)	
≥3	3 (15%)	1 (3.0%)	
Dysmenorrhea^a^	2.4 (±2.8)	6.8 (±3.0)	<.001^*∗*^
Deep dyspareunia^a^	.1 (±.4)	4.1 (±3.5)	<.001^*∗*^
Dyschezia^a^	0	3.2 (±2.5)	<.001^*∗*^

^*∗*^ *p* < .05. ^a^Visual analogic scale. ^b^Student's *t*-test. ^c^Pearson  *χ*^2^ test. Data presented as mean ± SD or numbers (%); DIE: deep infiltrative endometriosis.

**Table 2:** Comparison of AUC for menstrual CA-125, for midcycle CA-125 and for the difference and ratio between the two phases.

CA-125^a^	AUC	95% CI
Menstrual	0.96	0.87–0.99
Midcycle	0.89	0.78–0.96
Difference^a^	0.96	0.87–0.99
Ratio^b^	0.86	0.74–0.94

AUC: Area Under the Curve; 95% CI: confidence interval of 95%; ^a^CA-125 difference between menstrual and midcycle phases; ^b^CA-125 ratio between menstrual and midcycle phases.

**Table 3:** Comparison of controls and patients with DIE in menstrual and midcycle phases using 35 IU/ml as a cutoff value for CA-125.

CA-125	Controls (*n* = 20)	DIE (*n* = 34)	Total
Both negatives^a^	19	5	24
Both positives^b^	0	18	18
Menstrual positive only	1	11	12
Total	20	34	54

DIE: deep infiltrative endometriosis. ^a^In menstrual and in midcycle phases (CA-125 ≤ 35 IU/ml). ^b^In menstrual and in midcycle phases (CA-125 > 35 IU/ml); *χ*^2^ = 33,1 (*p* < .00001).
